# Accuracy of the novel digital non-cross-arch surgical guides with integration of tooth undercut retention and screw-bone support for implant placement in mandibular free-end

**DOI:** 10.1186/s12903-024-04329-z

**Published:** 2024-05-11

**Authors:** Qin Wu, Yuxin Lou, Jikui Sun, Chenyang Xie, Jiacheng Wu, Haiyang Yu

**Affiliations:** 1 State Key Laboratory of Oral Diseases & National Center for Stomatology & National Clinical Research Center for Oral Diseases, Department of Prosthodontics II, West China Hospital of Stomatology, Sichuan University, 14 Renmin South Road, 3rd Section, Chengdu, Sichuan 610041 China; 2 State Key Laboratory of Oral Diseases & National Center for Stomatology & National Clinical Research Center for Oral Diseases, Department of Dental Technology, West China Hospital of Stomatology, Sichuan University, 14 Renmin South Road, 3rd Section, Chengdu, Sichuan 610041 China

**Keywords:** Dental implant, Guided surgery, 3D printing, Distal free-end edentulism, Accuracy

## Abstract

**Background:**

Large cross-arch free-end surgical guides can obscure the visual field, compromising surgical accuracy due to insufficient stability at the free-end. This in vitro study aims to evaluate the accuracy of novel digital non-cross-arch surgical guides designed for implant placement at the mandibular free-end, incorporating tooth undercut retention and screw-bone support.

**Materials and methods:**

A mandibular dental model lacking left molars was utilized to fabricate unilateral (cross-arch) tooth-supported surgical guides (GT I, *n* = 20). Subsequently, two additional types of surgical guides were fabricated: GT II (covering two teeth, *n* = 20) and GT III (covering three teeth, *n* = 20). These novel surgical guides were designed to utilize the undercut of the supporting teeth for retention and enhance stability with screw-bone support at the guide’s free-end. Furthermore, 60 identical guiding blocks were assembled on the three types of surgical guides to facilitate the implants’ insertion. On a phantom head, 120 implant replicas were placed at the Federal Dentaire Internationale (FDI) teeth positions #36 and #37 on the dental model, employing a combination of surgical guides and guiding blocks. To assess accuracy, planned and placed implant positions were compared using intraoral optical scanning. Discrepancies in angulation and linear deviations, including the coronal/apical 3D deviations, lateral deviation as well as depth deviation, were measured. Statistical analysis was performed using two-way ANOVA and Bonferroni test (α = 0.05).

**Results:**

GT I exhibited significantly largest discrepancies, including angular and linear deviations at the crest and apex at every implant site. Especially in depth, at implant site #36, the mean deviation value of GT I (0.27 ± 0.13 mm) was twice as large as GT III (0.13 ± 0.07 mm), and almost twice as large as GT II (0.14 ± 0.08 mm). However, at implant site #37, this deviation increased to almost a five-fold relationship between GT I (0.63 ± 0.12 mm) and II (0.14 ± 0.09 mm), as well as between GT I and III (0.13 ± 0.09 mm). No significant discrepancies existed between the novel surgical guides at either implant site #36 or #37.

**Conclusion:**

This study provides a practical protocol for enhancing accuracy of implant placement and reducing the size of free-end surgical guides used at mandibular molar sites.

## Background

Accurate three-dimensional (3D) placement of dental implants is a key prerequisite for successful implant treatment [1, 2]. The adoption of prosthetically driven implant surgery is recommended, as it ensures favorable functional and biological outcomes [3, 4]. One effective approach to implement this concept is through static computer-assisted implant surgery (sCAIS), which helps reduce potential deviations associated with free-hand implant placement [5–8]. While the mean values of the implant deviations using sCAIS were found to be clinically acceptable, it is important to note that relatively large maximal deviations have been reported, and a safety margin of at least 2 mm should be respected [9–12].

To date, there is a consensus that the tooth-supported surgical guide exhibits higher accuracy compared to both the mucosa-supported and mixed supported surgical guides [13–15]. The mixed supported surgical guide is often used for directing implant placement in the distal free-end of missing molars [16, 17]. Intraoperatively, the unilateral tooth-supported surgical guide is subject to micromovement, bending, and tilting. These instabilities are due to the soft tissue supporting the free-end being resilient, resulting in larger deviations than the tooth-supported surgical guide [18–20]. An in vitro study using acrylic dental models indicated that the accuracy of sCAIS significantly decreased as the length of the free-end extension increased [21]. This is attributed to the more apparent tilting and bending effects of the guide, especially in posterior implant sites [10]. Generally, the guide cannot extend into the undercut area of the supporting teeth to achieve sufficient retention and stability [22]. To address these challenges, free-end surgical guides are usually configured in a cross-arch design by default, as more supporting teeth contribute to enhanced stability [16, 23]. To further mitigate instability, the ends of the surgical guide are sometimes connected in the form of a plate or rod, or fixation pins embedded in the bone are added [24, 25]. Although these measures can improve the accuracy of the free-end surgical guide, they unavoidably increase its size. A larger surgical guide not only occupies the limited oral space and obscures the visual field, but may also cause inconvenience to the surgeon and discomfort to the patient, especially in the mandible, due to the presence of the tongue. Moreover, additional costs and time are required for the production [21]. To address these drawbacks, we plan to improve the guide’s configuration, aiming to develop a novel no-cross-arch free-end surgical guide, with reduced dimensions. However, the absence of a cross-arch reduced the stability of the guide. To address this challenge, a principle derived from a removable partial denture (RPD) design was referenced, where enhancing retention improves denture stability [26]. The most direct method involves utilizing the undercuts on supporting teeth. Recently, a study reported remarkable improvement in the stability of a small four-teeth-supported surgical guide for single-tooth-gap cases by incorporating 0.1 mm buccal surface undercut on the supporting teeth [27].

Therefore, we designed a novel surgical guide that utilizes the undercut of 0.1 mm on both lingual and buccal surfaces of the supporting teeth to achieve self-retention. Additionally, a mini-screw supporting on the bone surface was added at the distal end of the surgical guide, preventing bending and tilting of the free-end. The above approach enhances the stability of the surgical guide, so it may be allowed to be designed in a small-sized form with only a few supporting teeth, but provides superior accuracy than a unilateral tooth-supported surgical guide with full-arch coverage.

Therefore, this study aimed to evaluate the accuracy of two novel surgical guides, one supported by two teeth and the other by three, with the goal of providing a strategy for downsizing the free-end guide. The null hypothesis was that the two types of novel surgical guides would have equal degree of accuracy and provide superior accuracy than the full-arch unilateral tooth-supported surgical guide.

## Materials and methods

### Model fabrication and scanning

**Fig. 1:**
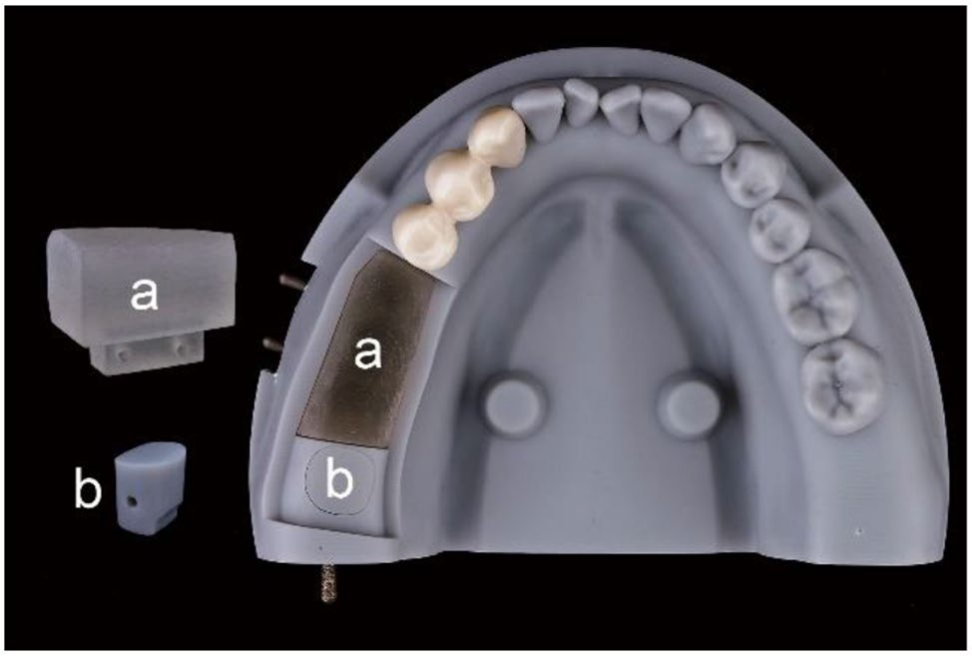
The dental model. (**a**) 3D printing resin implant bed. (**b**) Crown-free pedestal

A digital full-arch cast with regular mandibular dentition and cone-beam computed tomography (CBCT) data were obtained from a 35-year-old male patient. Using this digital data, a dental model excluding the left molars but incorporating a plug-in crown-free pedestal in place of the third molar was designed in an engineering software program (Geomagic Wrap 2015; 3D Systems). The model was then printed with DM15 photopolymer resin using a 3D printer (AccuFab-D1s; SHINING 3D). A replaceable resin module printed with VisiJet M3 Stoneplast photopolymer resin using another 3D printer (ProJet MJP 3600; 3D Systems), was used as implant bed to replace the first and second molars. The crown-free pedestal and implant bed could be fixed in the dental model using pins. In addition, to mimic the smooth surfaces of natural teeth, 1 mm thick zirconia crowns covered with enamel porcelain (SHTM; Aidite (Qinhuang Dao) Technology Co., Ltd.) were cemented using self-adhesive resin cement (RelyX U200; 3 M ESPE) on the three teeth adjacent to the edentulous area (Fig. 1). The complete dental model was scanned using an intraoral scanner (TRIOS 3, 3 shape) to acquire the standard tessellation language (STL) files.

### Implant planning and surgical guides fabrication

In an implant planning software (Blue Sky Plan 4.7; Blue Sky Bio), the STL files of the dental model were aligned with the CBCT data. Following the digital waxings from the full-arch cast, two 4.1 × 10 mm bone-level implants (Straumann BLT implant; Institut Straumann AG) were virtually positioned at the molar sites corresponding to the FDI teeth positions #36 and #37. The resulting model, incorporating the virtual implants, was then saved in STL format as a fiducial model.

In a dental software (exocad Dental CAD; exocad GmbH), two bottom models were created separately from the fiducial model: the first without any undercut and the second with 0.1 mm undercut. Subsequently, in Geomagic Wrap 2015, different types of surgical guides were designed based on the first and second bottom models.

**Fig. 2:**
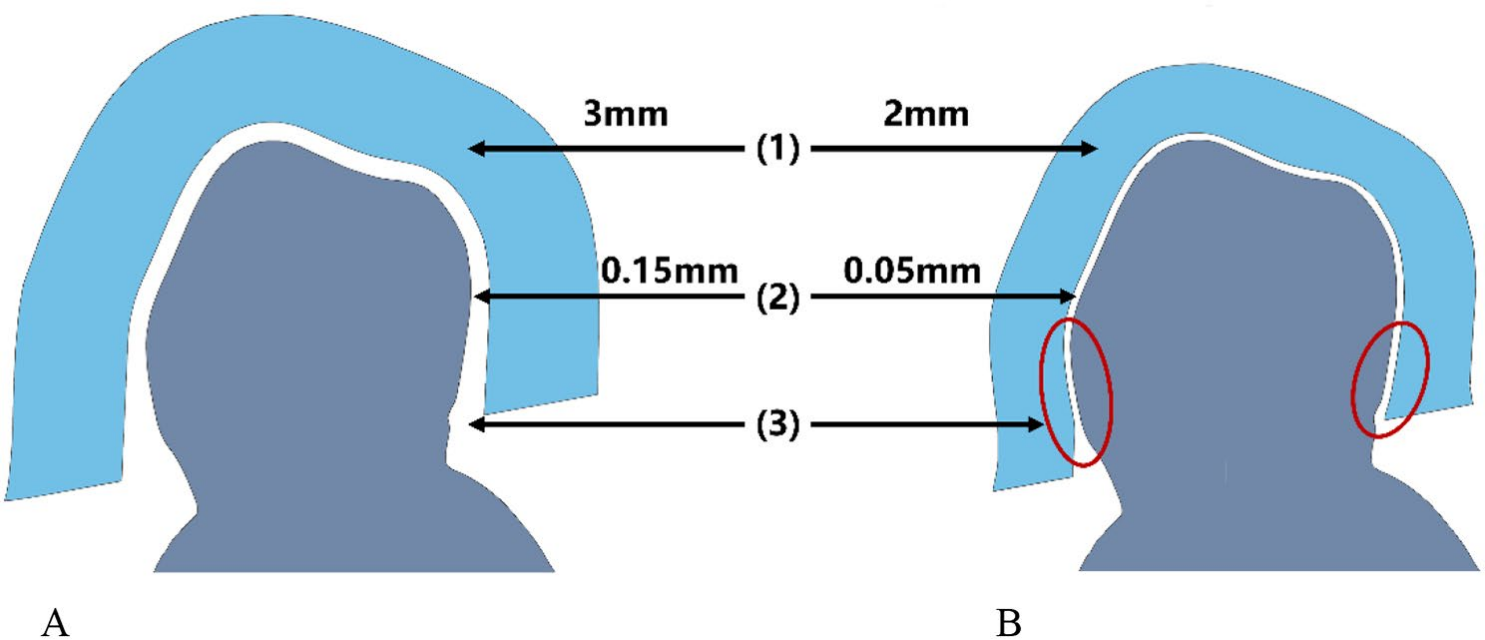
Schematic bucco-lingual cross-section comparing the design differences between the conventional surgical guide (GT I) (**A**) and the novel surgical guide (GT II, III) (**B**). (1) Reduced the surgical guide thickness from 3 mm to 2 mm. (2) Reduced the offset of guide-to-teeth from 0.15 mm to 0.05 mm. (3) Conventional surgical guide on the model with blocked undercuts, compared to novel surgical guide extends into undercut area (red ovals)

Using the first bottom model and the planned implants as a reference, a unilateral tooth-supported full-arch surgical guide (Guide Type I, GT I) was designed. It had a material thickness of 3 mm and a guide-to-teeth offset of 0.15 mm (Fig. 2A). In additional, a guiding block, attached to the surgical guide with pins, was designed for implant insertion.

Using the second bottom model in Geomagic Wrap 2015, two novel types of surgical guides were developed in two stages. First, a three-teeth-supported surgical guide (Guide Type III, GT III) was designed with a guide-to-teeth offset of 0.05 mm and a thickness of 2 mm (Fig. 2B). Subsequently, the GT III was trimmed to generate a two-teeth-supported surgical guide (Guide Type II, GT II).

**Fig. 3:**
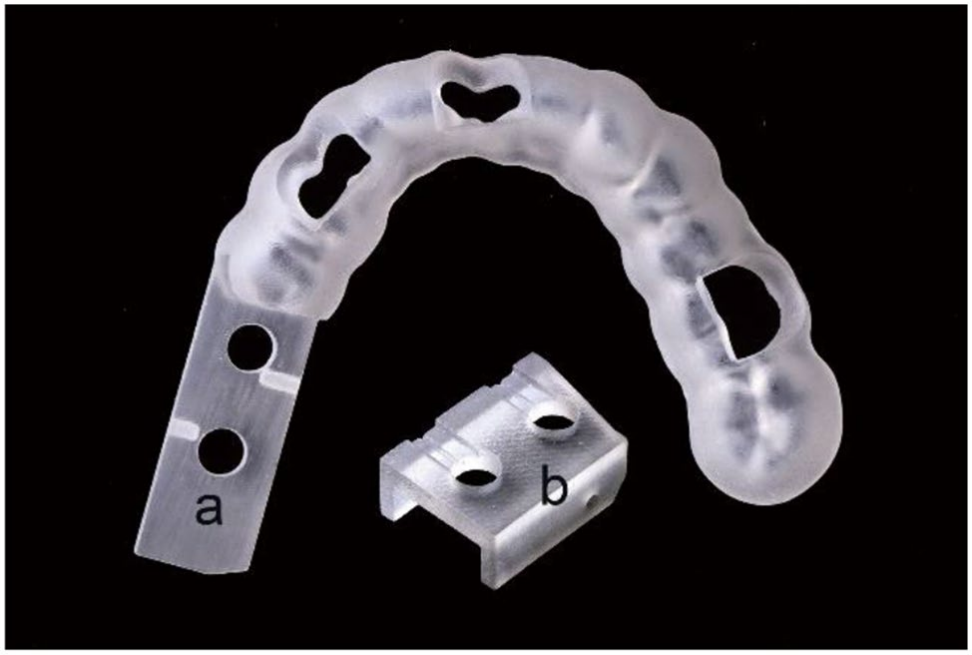
(**a**) Unilaterally full-arch supported surgical guide with free-end and (**b**) guiding block

**Fig. 4:**
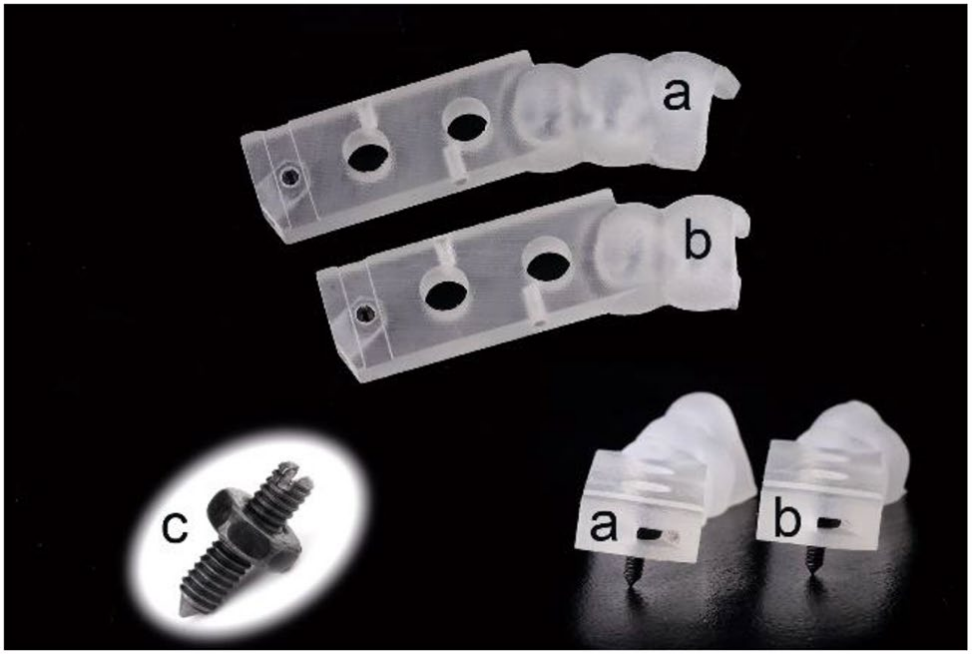
Novel surgical guide with a mini-screw and nut in free-end. (**a**) GT III covering three teeth. (**b**) GT II covering two teeth. (**c**) Mini-screw and nut

In addition to the aforementioned features, the distal end of the novel surgical guide was designed with a channel and hole to accommodate a mini-nut with an 8 mm long pointed screw (304 stainless steel, DIN934; Suzhou Qiangda Fastener Co., Ltd.). When the screw was threaded into the preset position and pressed against the bone surface, the upper end of the screw aligned with the upper plane of the hole. Finally, the above-designed surgical guides and guiding blocks were printed using ProJet MJP 3600 (Figs. 3 and 4). In total, 60 surgical guides (20 for each type of guide), 60 guiding blocks, 60 implant beds, and 60 crown-free pedestals were fabricated.

### Implant placement

**Fig. 5:**
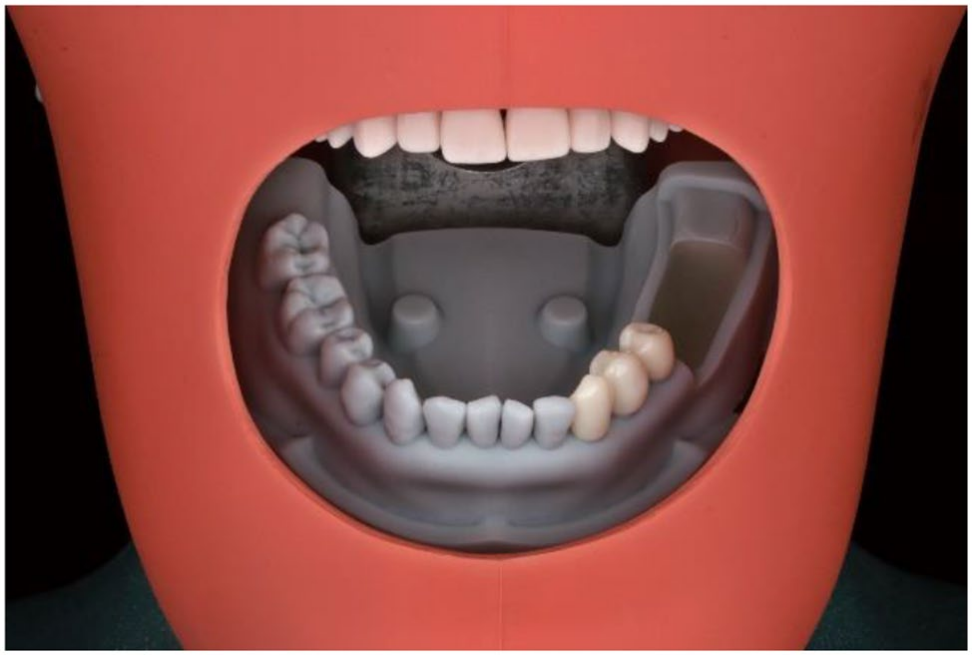
Dental model was mounted into a phantom head

**Fig. 6:**
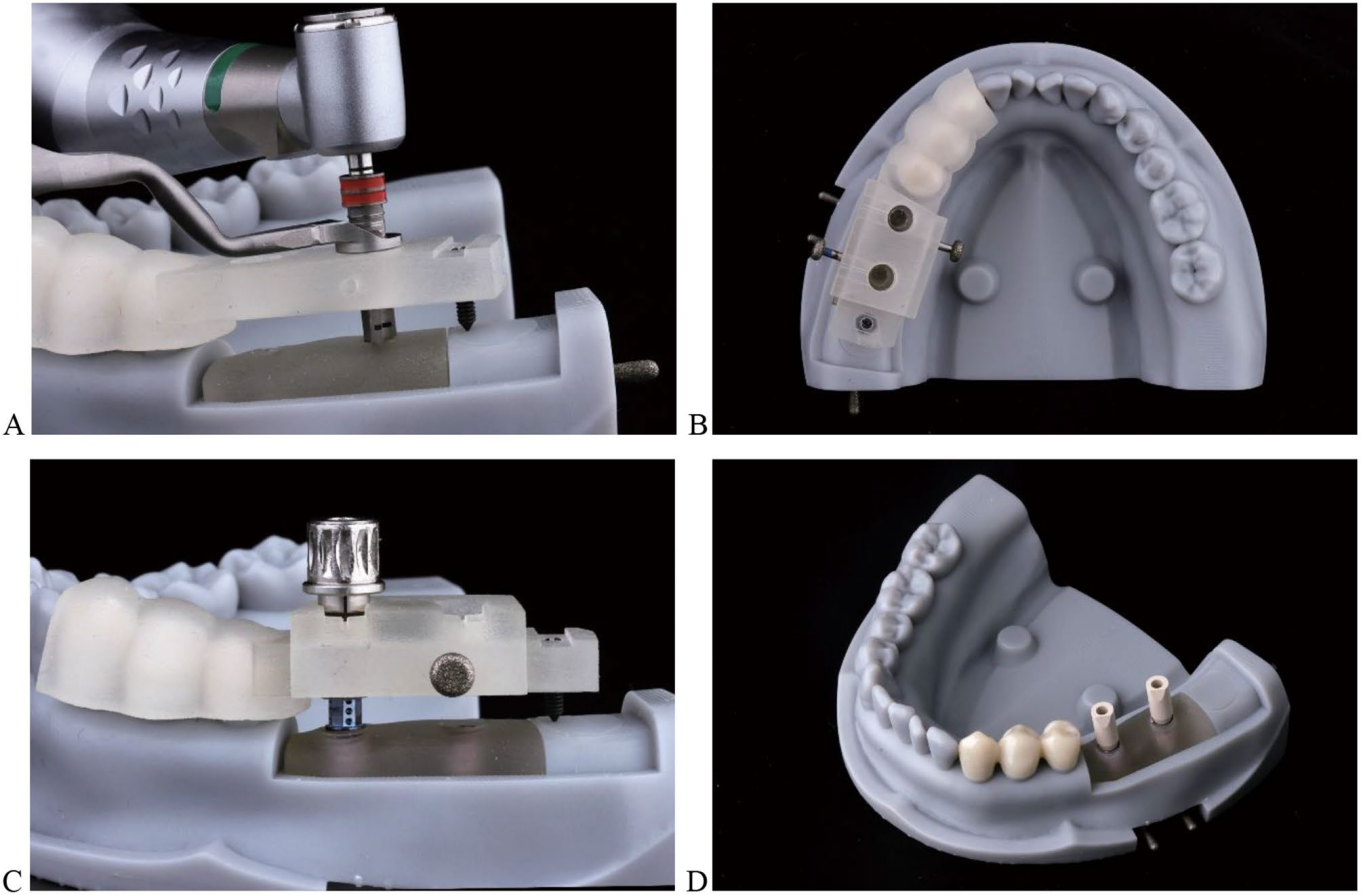
Detailed presentation of implant surgery guided by GT III. (**A**) Implant osteotomy preparation. (**B**) Fixed guiding block on surgical guide. (**C**) Inserted implants and aligned markings between GPA and guiding block. (**D**) Scanbodies were attached to implants

The implant bed and crown-free pedestal were fixed in the dental model using pins derived from the handles of discarded high-speed handpiece burrs (DIA-BURS; MANI Inc) (Fig. 1). Subsequently, the correct seating of each surgical guide was confirmed on the dental model via visual inspection. Implant surgery was simulated using the dental model mounted on a phantom head (Fig. 5). The Straumann Guided Surgery System for BLT implant was chosen for this study. 120 implant replicas (4.1 × 10 mm, Straumann BLT implant; Institut Straumann AG) were placed using a fully guided protocol executed by an operator with 10 years of experience in guided implant surgery. The surgical details were shown in Fig. 6.

### Data acquisition and accuracy analysis

**Fig. 7:**
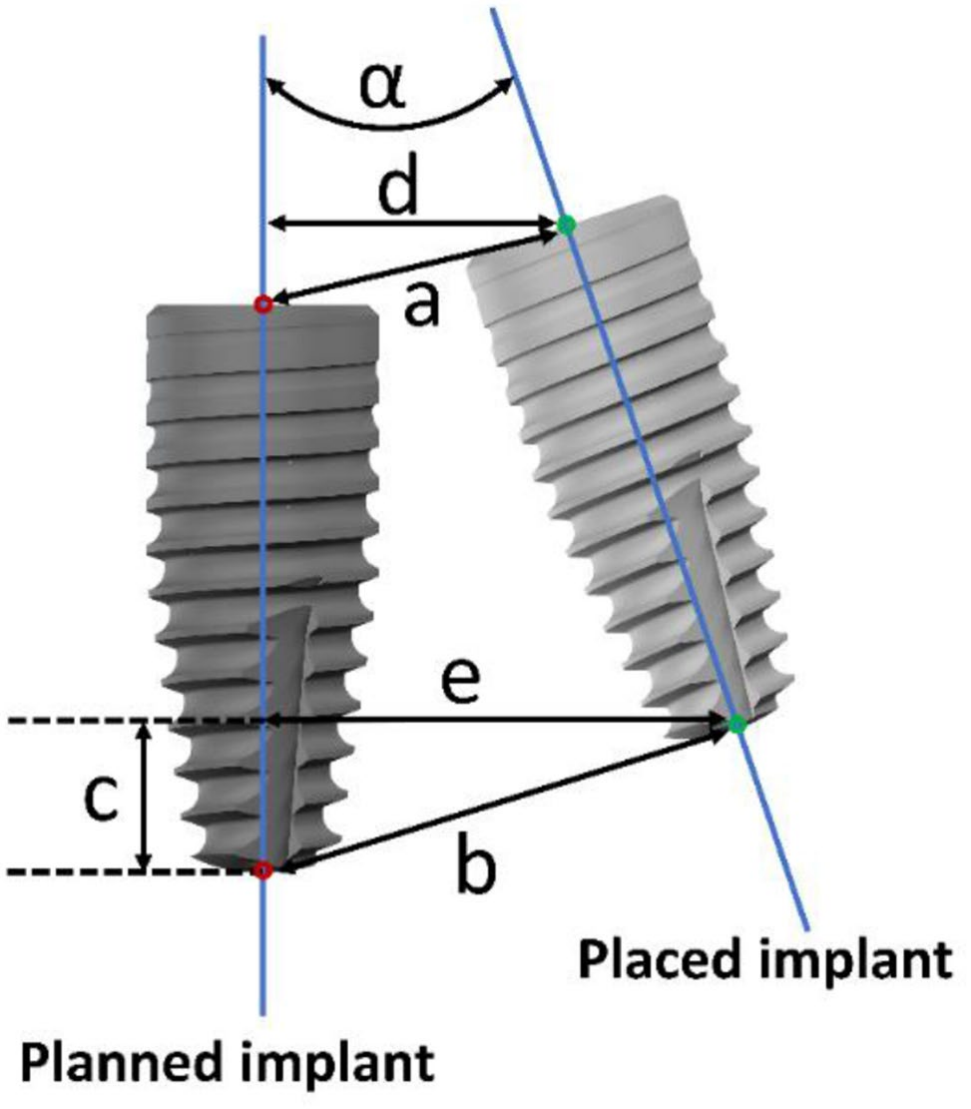
Measurements of deviations between planned and placed implant positions. (α) Angular Deviation. (**a**) Coronal 3D deviation. (**b**) Apical 3D deviation. (**c**) Depth deviation. (**d**) Coronal lateral deviation. (**e**) Apical lateral deviation

After the corresponding scanbodies (RC, Mono Scanbody; Institut Straumann AG) were attached to the implants (Fig. 6D), postoperative full-arch digital casts were obtained using TRIOS 3, and the data were saved as STL files. In Geomagic control 2015 software, a virtual RC scanbody from the exoCAD software database and a virtual 4.1 × 10 mm RC BLT implant from the Blue Sky Plan 4.7 software database were combined to form a registration unit. Subsequently, after superimposing the postoperative digital cast with the fiducial model in Geomagic control 2015, the registration units were registered into postoperative digital cast to record the placed implants for comparison with the planned implants. Two duplicate coordinate systems (CSYS) were established in each of the planned implant: the coronal center of the implant was set as the origin, the axis of the implant was set as the Z-axis and was positive to the coronal side, while the X-axis pointed distally. A schematic diagram of the deviations is shown in Fig. 7. Importantly, the coronal/apical deviation was decomposed into vertical (depth deviation) and lateral deviations according to the axis of the planned implant. All the above parameters were used in absolute value. Additionally, to show the dispersion of the coronal and apical centers, all the original data were also recorded as positive and negative, according to the CSYS. The depth deviation was positive when the placed implant is coronal to the planned implant or negative when apical to the planned implant.

### Statistical analysis

The results were assessed for normal distribution within groups using the Shapiro-Wilk test. Variance homogeneity was evaluated using Levene’s test. To examine the disparities between the planned and placed implant positions, a two-way ANOVA with Bonferroni multiple comparisons was separately conducted for each parameter. α was set at 0.05 for all tests. Boxplots were used to represent the measurements visually. 3D scatter plots were used to show the dispersion of coronal and apical centers of implants. The results were analyzed using statistical software (SPSS 27.0; IBM SPSS).

## Results

Totally, 120 implants were placed in 60 resin blocks with three types of surgical guides. For each implant site in each guide group, the deviations between the planned and placed implant positions are statistically analyzed. The angular deviation ranges for GT I, II, and III were 2.64–5.53°, 1.37–2.94°, and 1.28–2.70°, respectively. The ranges of the coronal 3D deviations for GT I, II, and III were 0.64–1.21 mm, 0.43–0.89 mm,0.31–0.88 mm, respectively. The ranges of apical 3D deviations for GT I, II, and III were 1.06–1.94 mm, 0.68–1.30 mm, and 0.57–1.15 mm, respectively. Detailed measurements for each type of guide at implant sites #36 and #37 are listed in Tables 11 and 2.

**Table 1:** 3D and angular deviations between the planned and placed implant positions

Implant Site	# 36			# 37			Total (*n* = 120)
Guide Type	GT I (*n* = 20)	GT II (*n* = 20)	GT III (*n* = 20)	GT I (*n* = 20)	GT II (*n* = 20)	GT III (*n* = 20)	
**Angular Deviation (°)**							
**Mean ± SD**	3.37 ± 0.53	2.03 ± 0.42	1.93 ± 0.41	4.63 ± 0.59	2.23 ± 0.45	2.14 ± 0.39	2.72 ± 1.08
**Min-Max**	2.64–4.34	1.37–2.85	1.28–2.61	3.49–5.53	1.46–2.94	1.46–2.70	1.28–5.53
**Median**	3.36	1.89	1.89	4.61	2.24	2.05	2.48
**95% CI**	3.12,3.62	1.83,2.23	1.74,2.12	4.35,4.90	2.02,2.44	1.96,2.32	2.52,2.92
**Coronal 3D Deviation (mm)**							
**Mean ± SD**	0.78 ± 0.10	0.60 ± 0.10	0.52 ± 0.09	0.99 ± 0.10	0.63 ± 0.13	0.59 ± 0.13	0.69 ± 0.19
**Min-Max**	0.64–0.95	0.43–0.78	0.33–0.65	0.79–1.21	0.44–0.89	0.31–0.88	0.31–1.21
**Median**	0.78	0.59	0.54	0.99	0.60	0.59	0.64
**95% CI**	0.73,0.82	0.55,0.65	0.48,0.57	0.95,1.04	0.57,0.69	0.53,0.65	0.65,0.72
**Apical 3D Deviation (mm)**							
**Mean ± SD**	1.33 ± 0.15	0.88 ± 0.13	0.82 ± 0.15	1.66 ± 0.17	0.97 ± 0.15	0.91 ± 0.13	1.10 ± 0.34
**Min-Max**	1.06–1.61	0.68–1.09	0.57–1.05	1.35–1.94	0.74–1.30	0.63–1.15	0.57–1.94
**Median**	1.34	0.87	0.84	1.67	0.93	0.92	0.99
**95% CI**	1.26,1.40	0.82,0.94	0.75,0.89	1.58,1.74	0.90,1.05	0.85,0.97	1.03,1.16

*3D* 3-dimensional, *CI* confidence interval, *SD* standard deviation

*#36* Mandibular left first molar, *#37* Mandibular left second molar, *GT I* Guide Type I, *GT II* Guide Type II, *GT III* Guide Type III

**Table 2:** Depth and lateral deviations between the planned and placed implant positions

Implant Site	# 36			# 37			Total (*n* = 120)
Guide Type	GT I (*n* = 20)	GT II (*n* = 20)	GT III (*n* = 20)	GT I (*n* = 20)	GT II (*n* = 20)	GT III (*n* = 20)	
**Depth Deviation (mm)**							
**Mean ± SD**	0.27 ± 0.13	0.14 ± 0.08	0.13 ± 0.07	0.63 ± 0.12	0.14 ± 0.09	0.13 ± 0.09	0.24 ± 0.21
**Min-Max**	0.01–0.55	0.03–0.29	0.01–0.28	0.39–0.83	0.00-0.38	0.01–0.33	0.00-0.83
**Median**	0.28	0.17	0.15	0.63	0.13	0.12	0.18
**95% CI**	0.21,0.34	0.10,0.18	0.10,0.17	0.57,0.68	0.10,0.18	0.09,0.17	0.20,0.28
**Coronal Lateral Deviation (mm)**							
**Mean ± SD**	0.71 ± 0.10	0.58 ± 0.10	0.50 ± 0.09	0.72 ± 0.16	0.61 ± 0.13	0.57 ± 0.13	0.62 ± 0.14
**Min-Max**	0.55–0.89	0.42–0.76	0.32–0.62	0.35–0.95	0.39–0.84	0.31–0.88	0.31–0.95
**Median**	0.73	0.59	0.50	0.77	0.59	0.57	0.61
**95% CI**	0.67,0.76	0.53,0.63	0.46,0.54	0.65,0.80	0.55,0.67	0.51,0.63	0.59,0.64
**Apical Lateral Deviation (mm)**							
**Mean ± SD**	1.30 ± 0.15	0.87 ± 0.13	0.80 ± 0.15	1.53 ± 0.18	0.96 ± 0.15	0.90 ± 0.13	1.06 ± 0.30
**Min-Max**	1.03–1.57	0.67–1.08	0.57–1.04	1.19–1.76	0.70–1.24	0.61–1.15	0.57–1.76
**Median**	1.33	0.86	0.83	1.55	0.92	0.89	0.98
**95% CI**	1.22,1.37	0.81,0.93	0.73,0.87	1.45,1.62	0.89,1.03	0.83,0.96	1.00,1.11

*3D* 3-dimensional, *CI* confidence interval, *SD* standard deviation

*#36* Mandibular left first molar, *#37* Mandibular left second molar, *GT I* Guide Type I, *GT II* Guide Type II, *GT III* Guide Type III

**Fig. 8:**
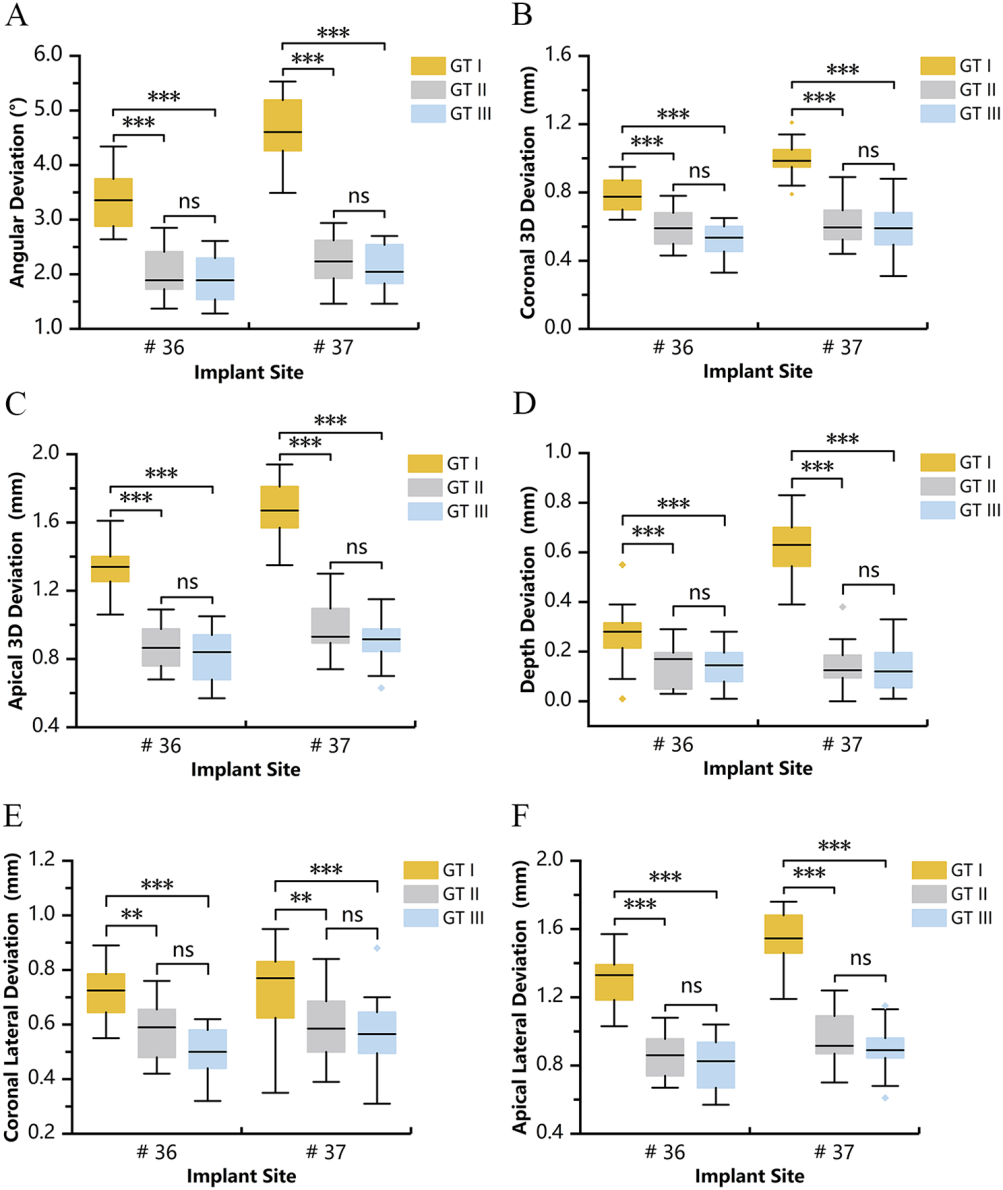
Box plots presented the deviations of implant placement using different guides at implant sites # 36 and # 37. (**A**) Angular deviation. (**B**) Coronal 3D deviation. (**C**) Apical 3D deviation. (**D**) Depth deviation. (**E**) Coronal lateral deviation. (**F**) Apical lateral deviation. Indicated as ** for *p* < 0.01, *** for *p* < 0.001, and ns for not significant, respectively

GT I demonstrated significantly larger mean values of angular and 3D deviations, both at the crest and apex for every implant site, when compared with the other two guide types (Fig. 8A, B,C). In the GT I group, except for the coronal lateral deviation, significant differences were found between implant sites #36 and #37 for each of the same parameters, and the deviations were larger at implant site #37. No statistically significant differences were found for any of the angular and linear deviations between GT II and III either at implant site #36 or #37 (Fig. 8). In the terms of apical 3D deviation in GT II group, the implants placed at implant site #37 (0.97 ± 0.15 mm) was slightly larger than that at implant site #36 (0.88 ± 0.13 mm), but statistically different (*p* = 0.048). Similarly, in GT III group, the apical 3D deviation was also statistically different between implant sites #36 (0.82 ± 0.15 mm) and #37 (0.91 ± 0.13 mm, *p* = 0.048).The other measurements between implant sites #36 and #37 were not significant either in the group GT II or III.

For depth deviation presented in Fig. 8D, the data was represented using absolute values. The depth deviations between GT II and III were not significant, while both were significantly different from GT I. At implant site #36, the mean depth deviation value of the GT I (0.27 ± 0.13 mm) was twice as large as GT III (0.13 ± 0.07 mm), and almost twice as large as GT II (0.14 ± 0.08 mm). At implant site #37, this deviation even increased to almost a five-fold relationship between GT I and II, as well as between GT I and III. The mean depth deviation values of 0.63 ± 0.12 mm, 0.14 ± 0.09 mm, and 0.13 ± 0.09 mm for GT I, II, and III, in that order.

With regards to coronal/apical lateral deviations, both GT II and III showed the second lowest and lowest deviation and were significantly different to GT I. The distributions of the lateral deviations of each implant site were presented in Fig. 8E, F.

**Fig. 9:**
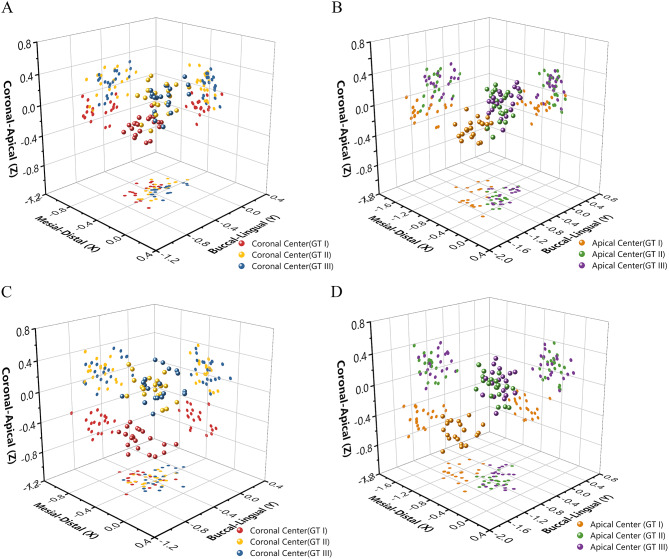
Three-dimensional scatter plot and the decomposition of coordinates. (**A**,**B**) Implant site #36. (**C**, **D**) Implant site #37

The dispersion of the coronal and apical centers of placed implants were shown in Fig. 9. The scatter of the centers casting shadows on the three planes showed that the implant positions of GT II and III were closer to the planned position than those of GT I. It is evident that the implants placed using GT I were more apically than those placed using the other two types of surgical guides. The implants deviated mesial and buccal sides regardless of the surgical guide, while this deviated tendency was more visible at the implant apex, especially in the GT I group.

## Discussion

The results revealed differences in accuracy when implants were placed at molar sites in the mandibular free-end using the three types of surgical guides. Both novel surgical guide types demonstrated higher accuracy than the full-arch guide (GT I), and they exhibited equal degree of accuracy. Therefore, the results support the null hypothesis.

In the sixth International Team for Implantology (ITI) consensus report, Daniel et al. [28] reported that the mean 3D deviation for sCAIS at the crest was 1.20 mm, at the apex was 1.50 mm and the angular deviation was 3.50°. In our study, the mean 3D deviations of 120 implants at the crest/apex were 0.69 ± 0.19 mm/1.10 ± 0.34 mm, and the mean angular deviation was 2.72 ± 1.08°, respectively. Given that this is an in vitro simulation of implant surgery on dental models, potential confounding parameters in clinical research can be ruled out. All variables were studied under near-ideal conditions, such as the supporting teeth on a model will not experience micro-movement under load conditions, unlike natural teeth. Accordingly, we can reasonably conclude that the results of this study are slightly superior to those previously reported. This discrepancy in outcomes might be attributed, in part, to the method of accuracy evaluation. We utilized optical impressions to obtain implant positions, a method proven to be more accuracy than that of using the CBCT scans [28–30].

The number of supporting teeth is an important factor that affecting surgical guide accuracy. A retrospective clinical study found that the optimal number of supporting teeth was approximately 10 [16], whereas Kholy et al. [21] found that a tooth-supported surgical guide with four teeth has accuracy equal to that of full-arch supported surgical guides. Therefore, our novel surgical guide was designed to be supported by at most three teeth, as at the free-end, a mini-screw supported on the bone surface could assume the supporting role of a tooth. Additionally, different from the previous reports, the novel surgical guides could be retained with the undercut of 0.1 mm on the supporting teeth. Therefore, the accuracy of the novel surgical guide did not decrease even when the number of supporting teeth was less than previously reported.

When encountering a distal extension situation during drilling, insufficient support at the free-end of guide can lead to micro-movement, tilting and bending, especially in the posterior region, resulting in significant deviations [31, 32]. Behneke et al. [19] suggested that using rigid materials to fabricate a guide with sufficient stiffness can prevent such instability. However, the cost will inevitably increase if more rigid materials, such as the metals, are selected. Alternatively, additional anchor pins or designed a rigid support at the cantilever can enhance guide stability [33]. Mai et al. [32, 34] reported that inserting an anchor micro-screw into the free-end of the alveolar ridge to support the surgical guide could enhance its stability and drilling accuracy. However, this technique may lead to increased bone trauma, as the screw needs to be placed in the alveolar crest before digital data collection and removed only after the surgery is completed, potentially interfered with the patient’s masticatory function during this period. An invitro study by Lin et al. [35] suggested that designing a strut supported on the bone surface at the free-end of a surgical guide could improve the accuracy of unilateral tooth-supported surgical guides. However, this technique requires a flap surgery on the distal alveolar ridge. Compared to the above two methods, our approach involved less trauma and a simpler operating procedure as the screw was pre-positioned in the surgical guide. During the guide placement, the sharp tip of the screw directly penetrated the mucosa to support on the bone surface. Furthermore, in this study, we observed that implants placed using the novel surgical guides (GT II and III) demonstrated significantly lower deviations than those placed using the control group surgical guides (GT I), especially in the terms of depth and lateral deviations. This observation further suggests that the mini-screw played a role in countering the instability of the guide’s free-end, similar to a supporting tooth.

The introduction of guide-to-teeth offset aimed to compensate for potential errors during image acquisition, data registration, and guide production, ensuring a correct fit between the surgical guide and supporting teeth [36]. Although larger offset values could improve the fit between guide and teeth, they tended to compromise the overall stability of the surgical guide [37]. Currently, the range of offset values found in literatures typically falls between 0.05 mm and 0.20 mm [37–39], with several studies adopting a 0.15 mm offset to design full-arch coverage surgical guides [21, 22, 40]. Moreover, the fabrication of small-sized surgical guide was more accurate than that of the large-sized surgical guide [41]. Therefore, in this study, we used a 0.15 mm offset for the full-arch surgical guide and a 0.05 mm offset for the novel surgical guides (covering two and three teeth), with the expectation that they would fit well on the teeth and that the novel surgical guides would effectively utilize the 0.10 mm deep undercut for stabilization. Surgical guides typically have a thickness range of 2–3 mm [21, 38, 39]. Therefore, we chose a thickness of 3 mm for the full-arch surgical guide and 2 mm for the novel surgical guides, allowing the novel surgical guides to enter the undercut with some elasticity. Based on the results of this study, the chosen parameter combination (0.05 mm offset and 2 mm material thickness) is deemed acceptable. However, it is necessary to conduct further investigation to determine if there are more optimal parameter combinations for the novel surgical guides.

Regarding the influence of sleeves on the accuracy of the surgical guide, Raabe C et al. [40, 42] reported that sCAIS using a sleeveless guide hole design demonstrated higher accuracy compared to sCAIS with the manufacturer’s sleeves. They attributed this difference to reduced tolerance, as the former had only two gaps (guide - key, and key - drill), but the latter contained three gaps (guide - metal sleeve, metal sleeve - key, and key - drill). In this study, we also utilized a sleeve-free guide design, which likely contributed to the comparatively favorable outcomes.

**Fig. 10:**
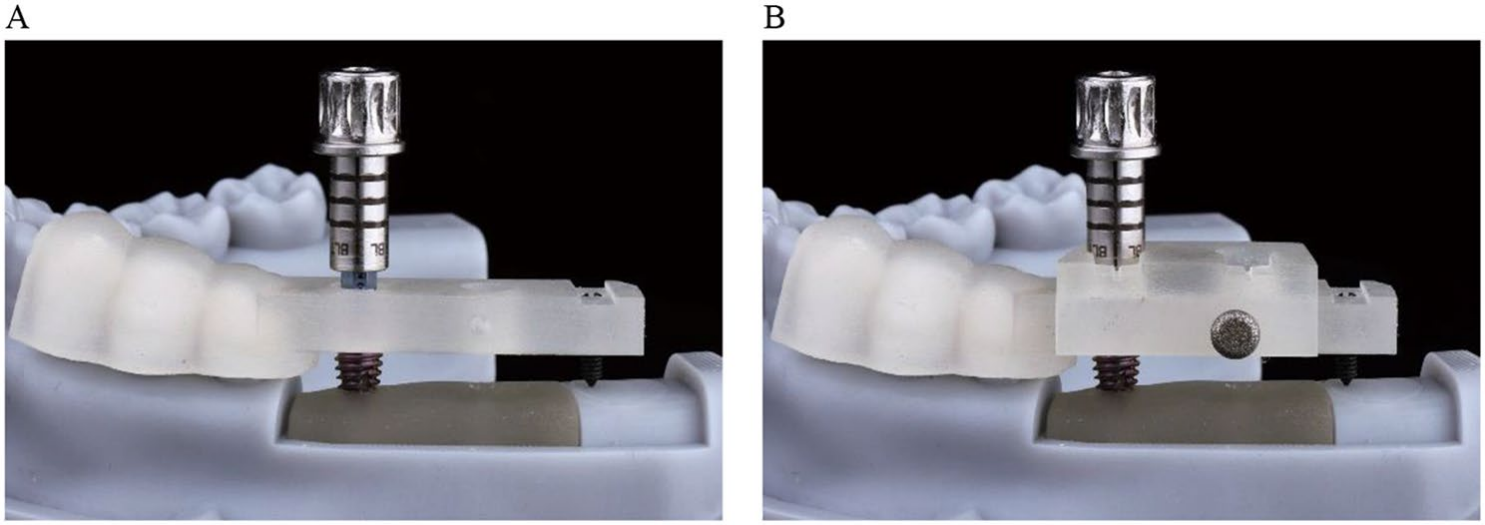
Function of the guiding block especially at beginning of implant insertion. (**A**) Without guiding block. (**B**) With guiding block

In this study we used the fully guided implant surgery protocol, which has superior accuracy compared to partially guided surgery [43, 44]. However, unlike previous reports, the insertion process of the implant was guided by the surgical guide and a guiding block (Fig. 6C). If without the guiding block, during the initial stage of implant insertion into the prepared hole, the guided portable adapter (GPA) did not make contact with the inner surface of the guide hole, so the entry path of the implant was not guided (Fig. 10A). Adding the guiding block effectively increased the length of the guide hole, ensuring that the implant was guided from the beginning of insertion (Fig. 10B). Based on geometric principles, as the length of the guide hole increases, the constraint of the guide hole on the GPA will be enhanced, leading to a reduction in deviations. In summary, the guidance of the implant insertion was improved in both time and space, resulting in lower deviations.

In a previous comparative study on the accuracy of sCAIS, Kim et al. [45]. found no significant difference between distal extension and tooth-end cases in their model study. However, a silicone artificial gingival material was used to cover the distal extension of the model in their study, which may provide some support to the free segment of the surgical guide and influenced their results. Therefore, the mucosal layer was excluded from our study to eliminate its potential effects on the free segment and solely focus on the role of the distal support mini-screw. This construction pattern for our model is similar to that of several previous studies [21, 35, 46].

Judging from current results and working conditions, it is emphasized that the indication of this approach is that the supporting teeth should be sufficiently stable, and provide at least 0.1 mm deep undercuts on both the buccal and lingual sides. At the same time, space is also required in the distal crest to place the support mini-screw. Furthermore, the current design of the novel surgical guide is only suitable for cases of missing molars. Subsequent studies should focus on improving its configuration to expand its range of application. For instance, determining the necessary extension length of the mesial part of the guide in cases where both molars and premolars are missing, and exploring the possibility of improving accuracy by adding more support min-screws in the distal part of the guide in such cases.

Smaller surgical guides not only reduce the cost and time of production, allowing more guides to be printed in a production cycle [21], but also provide more space for the surgeon to operate. Based on our results, we may have developed a method to reduce the size of free-end surgical guides, as the volumes of these novel surgical guides were smaller than those of the full-arch surgical guide and were within the range of clinical accuracy requirements. In this study, the volume of the full-arch surgical guide was 7346.39 mm^3^, whereas the volumes of the two novel surgical guides were 2234.61 mm^3^ (two supporting teeth) and 2575.49 mm^3^ (three supporting teeth). This represented reductions of 69.6% and 64.9%, respectively, compared to the full-arch surgical guide. In comparison to the upper jaw, the lower jaw has less space due to the presence of the tongue. A smaller surgical guide not only provides more spaces for the surgeon to operate, but also reduces the compression of the surgical guide on the tongue, which may help to reduce the patient’s discomfort during the operation. Therefore, it may be more logical to use a smaller surgical guide in the lower jaw. In light of the aforementioned considerations, the lower jaw was selected for this study. However, in the future we will also study the performance of these novel surgical guides in maxillary guided implant surgery, thereby enabling a comprehensive evaluation of their effectiveness.

Based on the results of this study, although the novel short surgical guides were beneficial in improving the accuracy of implant placement at molar sites in the free-end compared to the unilateral tooth-supported surgical guide covering full-arch, it is important to note that the distal supporting mini-screw with a sharp head will inevitably cause minor trauma to the mucosa and bone. Another limitation is the lack of the software for the automated generation of this novel surgical guide, resulting in a longer time required for its design. Finally, according to previous studies [47, 48], the accuracy of implant placement using digital surgical guides may vary between operators with different experience. Since this experiment was performed by the same operator, it is necessary to compare the results of using these novel surgical guides by different operators in further studies, as this will not only help to demonstrate their general applicability, but also provide a reference for further optimization of the design and the development of the training programs for the use of these novel surgical guides. After this, preclinical and clinical randomized controlled trials are needed to verify the effectiveness and accuracy of this novel design, and provide a basis for developing relevant design software.

## Conclusions

Within the limitations and results of this in vitro study, the following conclusions were drawn:

1. The novel surgical guides demonstrated higher accuracy compared to unilateral tooth-supported surgical guides with full-arch coverage.
2. The deviations of the implants placed using the novel guides were within the clinically acceptable range.
3. In the case of missing molars in the mandibular free-end, the protocol using the undercut of supporting teeth for surgical guide retain, along with screw-bone support at the free-end of the surgical guide, could shorten the guide and enhance the accuracy of the unilateral tooth-supported surgical guide. This improvement may result from increased the stability and reduced the bending and tilting of the surgical guide.
4. This study provides a practical protocol for enhancing accuracy of implant placement and reducing the size of free-end surgical guides used at mandibular molar sites.

## Data Availability

All essential data of this study is presented in the manuscript. The data of this study are available from corresponding author on reasonable request.

## Abbreviations

GT: Guide Type
FDI: Federal Dentaire Internationale
3D: Three-Dimensional
sCAIS: Static Computer-Assisted Implant Surgery
RPD: Removable Partial Denture
CBCT: Cone-Beam Computed Tomography
STL: Standard Tessellation Language
CSYS: Coordinate Systems
GPA: Guided Portable Adapter
